# Link between iron-mediated lipid peroxidation and polycystic ovary syndrome (PCOS): exploring the genes underlying iron regulatory mechanism

**DOI:** 10.1186/s13048-024-01562-6

**Published:** 2025-03-08

**Authors:** Nighat Hayat, Zertashia Akram, Nayab Khalid, Nasreen Rehmat Ullah, Tehmina Mazhar

**Affiliations:** 1 Cancer Genetics and Epigenetics Lab, Department of Biosciences, COMSATS University, Islamabad, Pakistan; 2 Department of Gynecology, KRL Hospital, Islamabad, Pakistan

**Keywords:** PCOS, Lipid peroxidation, Iron overload, Oxidative damage, Ferroptosis, Antioxidants, Rotterdam criteria

## Abstract

**Objective:**

Mechanism underlying the etiology of polycystic ovary syndrome (PCOS) is still debatable. Present study explores the link between iron-mediated ferroptosis and PCOS.

**Methodology:**

Blood samples were collected from 150 PCOS females along with healthy controls. Expression analysis of FTH1, NCOA4, GPX4, HAMP, A2M and HP genes was estimated by RT-qPCR. Serum was used for estimation of lipid peroxidation, peroxidase enzyme, ferritin and total protein.

**Results:**

Relative expression of FTH1 (*P* < 0.05), HAMP (*P* < 0.01), GPX4, A2M, HP (*P* < 0.001) was downregulated and NCOA4 (*P* < 0.001) was upregulated in PCOS group compared to control. A significant difference was observed in mRNA expression of selected genes when ≤ 30year age group PCOS was compared to > 30year age PCOS group and their respective controls. Deregulation of gene expression was prominent in PCOS group with obese and overweight BMI compared to underweight and normal BMI group. Menstrual cycle length and marital status of PCOS females had no significant association with selected gene expression. Expression deregulation in targeted genes was observed in PCOS patients with complaints of either diabetes, high blood pressure or both. Increased level of lipid peroxidation, serum ferritin and total protein, while decreased peroxidase activity was observed in PCOS group (*P* < 0.001) compared to control.

**Conclusion:**

The present study postulated the role of iron overload in trigger of ferroptosis following elevated lipid peroxidation and low peroxidase activity. Moreover, unveil the association of genes related to iron-regulating metabolism with etiology of underlying PCOS mechanism.

**Graphical Abstract:**

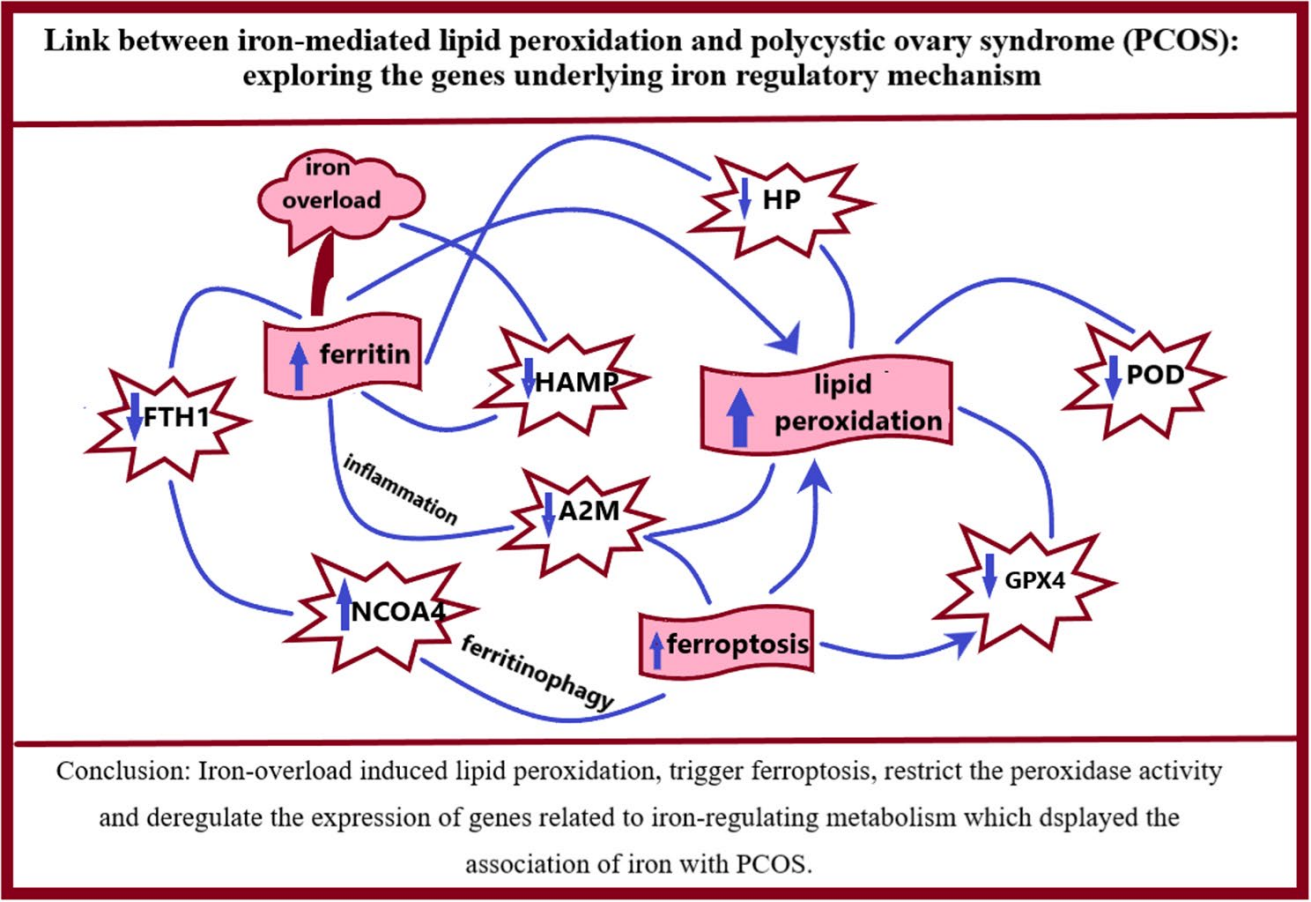

## Take home message

Present study will be helpful to explain pathway that why ferritin levels will elevated in PCOS patients and also exploring the cause of PCOS with reference to iron-overload.

## Introduction

Polycystic ovary syndrome (PCOS) is a multifactorial reproductive/endocrine disorder with worldwide prevalence of 5–10% in females of reproductive age (18–45 years) [1]. However, South Asia community presented worst condition. Pakistani population contained 52% females with complaints of PCOS [2]. The pathophysiology of PCOS is complex and it can occur at any stage of reproductive life [3]. PCOS interacts with multiple factors including genetics, environmental, fetal and metabolic and can be associated with hyperandrogenism, hirsutism, anovulation, cystic appearance of ovaries insulin resistance, oxidative stress, chronic inflammation and cardiovascular diseases [4, 5]. Correlation between PCOS and impaired programmed cell death has been reported. Cell death is highly regulated mechanism, and recycling is important for life sustaining processes. Therefore any dysregulation can lead to the trigger of many diseases [6–8].

Ferroptosis was discovered in 2012 as a non-apoptotic iron dependent cell death, characterized by elevated lipid peroxidation [9]. Various factors such as free iron, depleted antioxidants, increased ROS production substantially lipid ROS can activate ferroptosis [10]. Iron is a redox active element therefore, it is worth to maintain iron homeostasis. Iron maintenance, utilization, absorption or recycling are critical for cellular function. Imbalanced homeostasis promotes accumulation of iron, ROS production, cell damage which ultimately leads to ferroptosis [11, 12]. The underlying mechanism is still ambiguous but researchers believe to explore the link between iron and reproductive health. Interestingly, iron acted like a double-edged sword as iron deficiency or iron overload, both conditions would pinch the female fertility [13]. Consequently, iron overload would induce various reproductive dysfunctions including pre-eclampsia, endometriosis and pregnancy problems [14]. Iron accumulation and oxidative stress would affect ovarian follicles of all stages including microenvironment of follicles hence playing a pivotal role in pathogenesis of PCOS or may led to impaired female fertility, although interception of iron overload might manage the condition of PCOS [15–17]. Elevated iron levels were observed in peritoneal cavity and in fimbriae of fallopian tube. Through Fenton reaction iron gets engaged in production of ROS and can trigger ferroptosis [18]. Therefore, iron toxicity in concordance with oxidative stress induced negative impact on female fertility [13]. To regulate redox balance the body has an efficient system of antioxidant enzymes including SOD, CAT and GPX4. GPX4 is the gatekeeper of ferroptosis, neutralize oxidative species and detoxify the impact of ROS [19, 20].

Various proteins are incriminated in iron storage, transportation and export within the body [21]. The earliest protein is ferritin which acts as body reservoir /storage for iron. High ferritin levels were observed in oxidative stress and inflammatory conditions [11]. Hepcidin protein encoded by HAMP gene is a critical regulator of iron metabolism. Hepcidin inhibits iron absorption, restricts iron export by forming a complex with ferropotin (FPN) protein [22]. HAMP will lower down free iron in circulation by intracellular removal [23]. NCOA4 is the moderator in iron metabolism, interacting directly with the heavy chain of ferritin (FTH1). It promotes DNA damage, hence gets involved in ferritinophagy and enhances the release of free iron [8]. Haptoglobin (HP) protein inhibits oxidative activity of free hemoglobin hence acting as an antioxidant and anti-inflammatory agent [24]. Alpha-2 macroglobulin (A2M) is a proteinase inhibitor, plays its role in homeostasis, impedes various proteinases inculpated in coagulation [25] including thrombin, trypsin, and collagenase by a special trapping technique [26]. Moreover, A2M being a member of the Globulin family can perform other functions in our body like acting as anti-inflammatory agent to control inflammation and protective against infections [27]. The mechanism underlying the involvement of iron overload in progression of PCOS is still under the cover. To date little is known about the collusion of ferroptosis in PCOS. The objective of this study is to investigate the role of iron-mediated ferroptosis in the etiology of polycystic ovary syndrome (PCOS) by analyzing the expression of specific iron metabolism-related genes and assessing associated biochemical markers in affected individuals. This includes evaluating the expression levels of FTH1, NCOA4, GPX4, HAMP, A2M, and HP in blood samples from PCOS patients compared to healthy controls. Additionally, the study aims to explore how factors such as age, body mass index, and comorbid conditions influence gene expression and biochemical profiles, specifically lipid peroxidation, serum ferritin, and peroxidase activity. Ultimately, the study seeks to elucidate the connection between iron overload and ferroptosis in the context of PCOS, contributing to a better understanding of the underlying mechanisms of this condition.

## Methodology

### Study design

Present study comprised two groups of females one is healthy control and other is clinically diagnosed females with PCOS. Samples were collected with prior permission of each participant. A unanimous questionnaire was filled containing information about routine, medical, family history and body weight of every member. The present study was approved by Ethical Review Committee of COMSATS University Islamabad (CUI/Bio/ERB/2023/29). PCOS patients were further categorized into different groups based on age, body mass index (BMI), menstrual cycle length, bleeding pattern, marital status and complaints of other diseases.

### Inclusion/exclusion criteria

Our study was not a cohort-based study. Reproductive age (17-42years) females were included in this study. Pregnant and menopause females were excluded from this study. For patient group, only females with clinical diagnosis of PCOS based on Rotterdam criteria were included, while females with other reproductive disorders were excluded. Blood samples were collected randomly at any day of menstrual cycle as the studied population of PCOS comprised females with different cycle length, duration and bleeding pattern. PCOS females with history of other diseases like diabetes, kidney illness, high blood pressure was included in this study. For control group, healthy females with regular menstruation and no other complaint of any reproductive disorders were included in this study.

### Blood sample collection

Blood samples of PCOS patients (*n* = 150) were collected along with age and gender matched healthy control females from different hospitals. Blood was collected in vacutainer and stored at 4 °C. Serum was separated from whole blood and stored at -20^o^C for further analysis.

### Expression analysis

RNA was extracted from whole blood of control and PCOS patients by Trizol reagent method [28]. Extracted RNA was quantified using Nanodrop spectrophotometer (ND-100, USA) and best quality RNA was converted to cDNA using RevertAid First Strand cDNA Synthesis Kit (Thermo Scientific, USA). Primers of selected genes (FTH1, NCOA4, A2M, HAMP, HP, GPX4) and housekeeping gene ACBT were designed using IDT (Table 1). Expression of genes were determined using RT-qPCR (Applied Biosystem). mRNA expression of selected and housekeeping gene was calculated by 2^− delta−delta CT^ method.

**Table 1:** Primer sequence of selected and housekeeping genes

Gene	Primer a	Sequence
HAMP	Forward	5’CACAACAGACGGGACAACTT3’
HAMP	Reverse	5’CGCAGCAGAAAATGCAGATG3’
GPX4	Forward	5’TTCCTCATCGACAAGAACGG3’
GPX4	Reverse	5’ACTTGTGGAGCTAGAAATAGTGG3’
NCOA4	Forward	5’GTTTGTGATCTCTTTGCCTGTATG3’
NCOA4	Reverse	5’ATTCCCAACGGTTACATCTTGA3’
FTH1	Forward	5’CCCCCATTTGTGTGACTTCAT3’
FTH1	Reverse	5’GCCCGAGGCTTAGCTTTCATT3’
A2M	Forward	5’GAAATCAGGTGGAAGGACAGAG3’
A2M	Reverse	5’AGGCCACACACTGATACATTC3’
HP	Forward	5’CCAGGTAGATATTGGGCTCATC3’
HP	Reverse	5’ACGCCCTACTTCTGCATAATC3’
ACBT	Forward	5’TTCTCTGACCTGAGTCTCCTT3’
ACBT	Reverse	5’ACACCCACAACACTGTCTTAG3’

### Biochemical assays

#### Peroxidase activity (POD)

Peroxidase activity was measured in serum samples of control and PCOS patients using a previously described method [29] with slight modifications. The reaction mixture was prepared using a mixture of 50mM phosphate buffer (pH 5.0), 20mM guaiacol, 40mM hydrogen peroxide and 0.1 ml serum sample. The reaction mixture was poured in the cuvette and absorbance was measured at 470 nm at interval of 1 min by spectrophotometer. POD activity is described as an absorbance change of 0.01 unit/min.

#### Lipid peroxidation/ (TBARS assay)

Lipid peroxidation in serum samples of control and PCOS patients were measured using thiobarbituric acid reactive substances (TBARS) assay. Principle of TBARS based on formation of complex between thiobarbituric acid (TBA) and MDA in serum samples. Reaction mixture (0.1 M phosphate buffer, 100 mM ascorbic acid, 0.1 ml serum sample) was prepared and incubated at 37 °C. To stop the reaction, 10% tricholoroacetic acid (TCA) was added to the reaction mixture. Afterwards 0.67% TBA was added to each sample and was boiled in water bath for 15 min to promote the TBARS formation. After cooling the samples on crushed ice, samples were centrifuged at 2500×g for 10 min and supernatant was collected. Absorbance was measured at 532 nm by spectrophotometer. The molar extinction coefficient of 1.56 × 105 M − 1 cm − 1 was used to calculate results as mM TBARS/mg protein at 37 °C.

#### Ferritin estimation

Serum ferritin level was estimated in control and PCOS group using using human specific Ferritin ELISA kit (Bio-active Diagnostic Systems, Voehl/Germany), read by microplate reader (Platos R496, AMP Diagnostics) through standard curve method. Reference range of the kit was 10-100ng/ml. Minimum detection value of ferritin was 1ng/ml.

#### Total protein estimation

Total protein (albumin + globulin) was estimated in serum samples of control and PCOS patients using commercially available total protein kit (Bio-Active-Diagnostic-System, Voeh1Germany). The kit was based on the principle of biuret reaction. Readings were taken at 546 nm on spectrophotometer. Minimum sensitivity for total protein detection was 0.17 g/dl. Reference range was 6.6–8.7 g/dl.

### Statistical analysis

Relative expressions of selected genes including HAMP, GPX4, FTH1, NCOA4, HP, A2M were compared between healthy control subjects and PCOS patients using student t-test. For comparison between multiple groups, One Way ANOVA was used following post ANOVA multiple comparison Tukey’s test. Serum ferritin, protein, POD and TBARS were compared between control and PCOS patients using student t-test. Cycle length was compared between PCOS patients by student t-test. PCOS group was compared based on BMI using One-Way ANOVA following post ANOVA multiple comparison Newman-Keuls test. Expression GPX4 versus POD, GPX4 versus TBARS and POD versus TBARS were compared using Pearson correlation coefficient. The whole data was analyzed using GraphPad Prism. Level of significance was considered as *P* < 0.05.

## Results

Present study comprised total 300 female participants with 150 were healthy controls and 150 were PCOS diagnosed patients. Demographic details of control and PCOS group are presented in Table 2.

**Table 2:** Demographic details of females from control and PCOS group

Parameters	Control (*n* = 150)	PCOS (*n* = 150)
Mean age (year)	32.98 ± 2.13	30.19 ± 1.02
≤ 30year	74(49.33%)	83(55.33%)
> 30year	76(50.66%)	67(44.66%)
Mean body weight	64.21 ± 2.13	67.63 ± 3.26
≤ 65 kg	83(55.33%)	45(30%)
> 65 kg	67(44.66%)	105(70%)
Married	90(60%)	116(77.33%)
Unmarried	60(40%)	34(22.66%)
Miscarriage	17(11.33%)	101(67.33%)

Demographic parameters including menstrual cycle length, bleeding pattern, body mass index, medical history of PCOS patients was recorded.

**Fig. 1:**
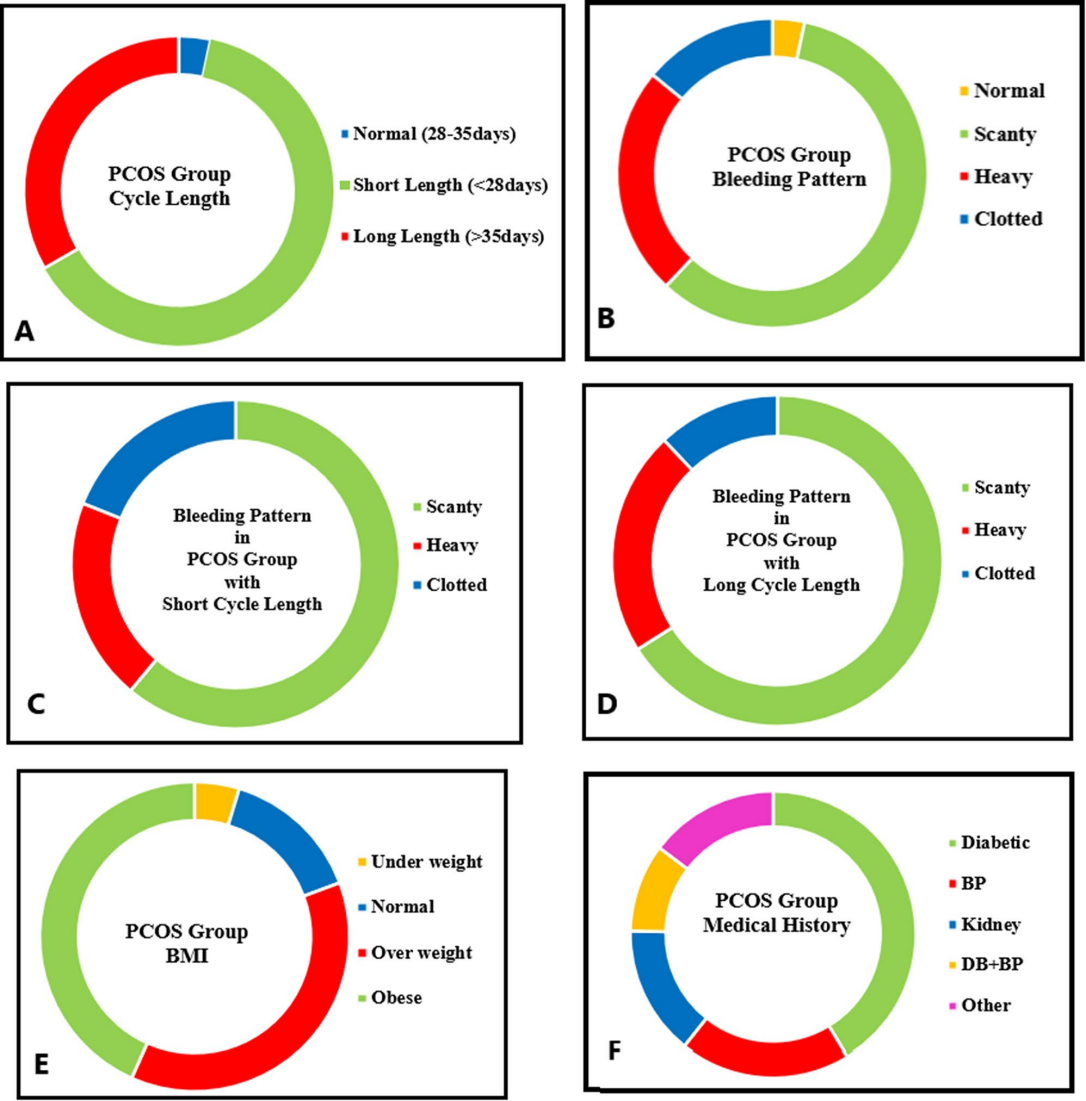
Demographic details showing distribution of females with PCOS including menstrual cycle length (**A**), overall bleeding pattern (**B**), bleeding pattern with short cycle length (**C**), bleeding pattern with long cycle length (**D**), body mass index (BMI) (**E**), medical history of having other diseases (**F**)

Three sub-groups were formed for PCOS patients based on menstrual cycle length: PCOS with normal cycle length (28–35 days), with short cycle length (< 28 days) and with long cycle length (> 35 days). Among 150 PCOS patients, fewer number of females (3.33%) showed normal cycle length, while majority of PCOS females (63.33%) presented with short cycle length. (Fig. 1A).

Based on blood discharge, four bleeding patterns (normal, scanty, heavy, clotted) were observed in PCOS patients. In 150 PCOS patients, fewer females had normal bleeding (3.33%), major number (58.66%) showed scanty blood discharge, while 24.0% with heavy bleeding and 14.0% presented with clotted bleeding (Fig. 1B).

PCOS patients with normal cycle length showed a normal pattern of bleeding. While, PCOS patients with short cycle length showed different bleeding pattern. About 61% females showed scanty bleeding, 20% with heavy bleeding and 18% showed clotted pattern of bleeding discharge (Fig. 1C). Similarly, in PCOS females with long cycle length, 66% presented scanty bleeding, 22% with heavy bleeding and only 6% with clotted bleeding pattern (Fig. 1D).

Four sub-groups were designed for PCOS patients based on their body mass index as females with < 18.5BMI (underweight), with 18.5-24.9BMI (normal), with 25.0-29.9BMI (overweight) and females with ≥ 30BMI (obese). More number of females was observed group of ≥ 30BMI and least number (4.66%) was observed in group of < 18.5 BMI. Only 14.66% females were found in 18.5-24.9BMI group and 37.33% were in 25.0-29.9BMI (Fig. 1E).

Based on medical history, PCOS patients had complaints of other diseases as well. In 150 PCOS females, 41.33% patients had complaint of diabetes (41.33%), 19.33% had high blood pressure, 14.66% with complaint of kidney illness and 10% were reported with diabetes along with high blood pressure. Although 14.66% PCOS females had complaint of illness other than these diseases like stomach issue, arthritis, inflammation (Fig. 1F).

### Relative expression of selected genes

**Fig. 2:**
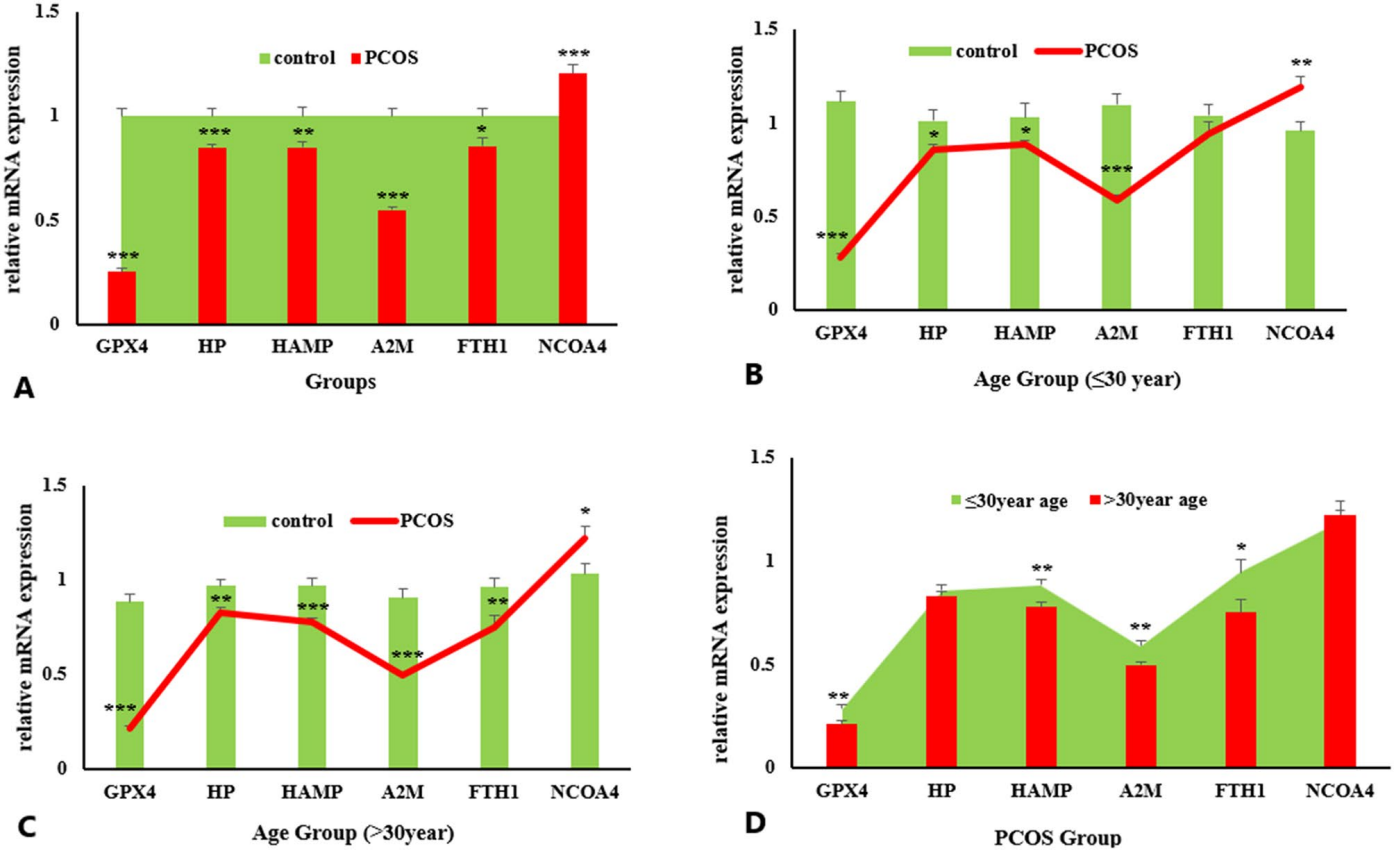
Relative expression of selected genes (GPX4, HP, HAMP, A2M, FTH1, NCOA4) in control and PCOS group showing comparison; control versus PCOS group (**A**), ≤ 30year age PCOS versus ≤ 30year control (**B**), > 30year age PCOS versus > 30year control (**C**), ≤ 30year age versus > 30year PCOS (**D**). Data was expressed as Mean ± SEM. Data was compared using One-Way ANOVA followed by multiple comparison Tukey’s test. *P* < 0.05*, *P* < 0.01**, *P* < 0.001***

Relative mRNA expressions of genes related to iron metabolism and oxidative stress were estimated PCOS patients compared to control group. mRNA expression of GPX4, HP, A2M (*P* < 0.001), HAMP (*P* < 0.01) and FTH1 (*P* < 0.05) were significantly downregulated in PCOS group compared to control. However, expression of NCOA4 was significantly (*P* < 0.001) upregulated in PCOS group compared to control group (Fig. 2A).

### Age

PCOS and control group was further categorized into two sub-group based on mean age as ≤ 30 year and > 30year aged women. Relative expression of GPX4, A2M (*P* < 0.001), HP and HAMP (*P* < 0.05) were significantly decreased and NCOA4 expression was significantly increased (*P* < 0.01) in ≤ 30year age PCOS group compared to control of same age group. No significant difference was observed in the expression of FTH1 gene between control and PCOS group (Fig. 2B).

In > 30year age group, relative expression of GPX4, HAMP, A2M (*P* < 001), HP and FTHI (*P* < 0.01) was significantly downregulated while NCOA4 was upregulated (*P* < 0.05) in PCOS patients compared to healthy control subjects of same age group (Fig. 2C).

Relative expression of selected genes was compared between ≤ 30year versus > 30year age group of PCOS females. mRNA expression of GPX4, HAMP, A2M (*P* < 0.01) and FTH1 (*P* < 0.05) gene was significantly decreased in PCOS female of > 30year age compared to PCOS female of ≤ 30year age. Whereas non-significant difference was observed in expression of HP and NCOA4 gene (Fig. 2D).

### PCOS group

PCOS patients were further categorized into various sub-groups considering their demographic details including menstrual cycle length, BMI, miscarriage history, complaints of other diseases and relative expression of selected genes was compared in all groups.

### Menstrual cycle length

**Fig. 3:**
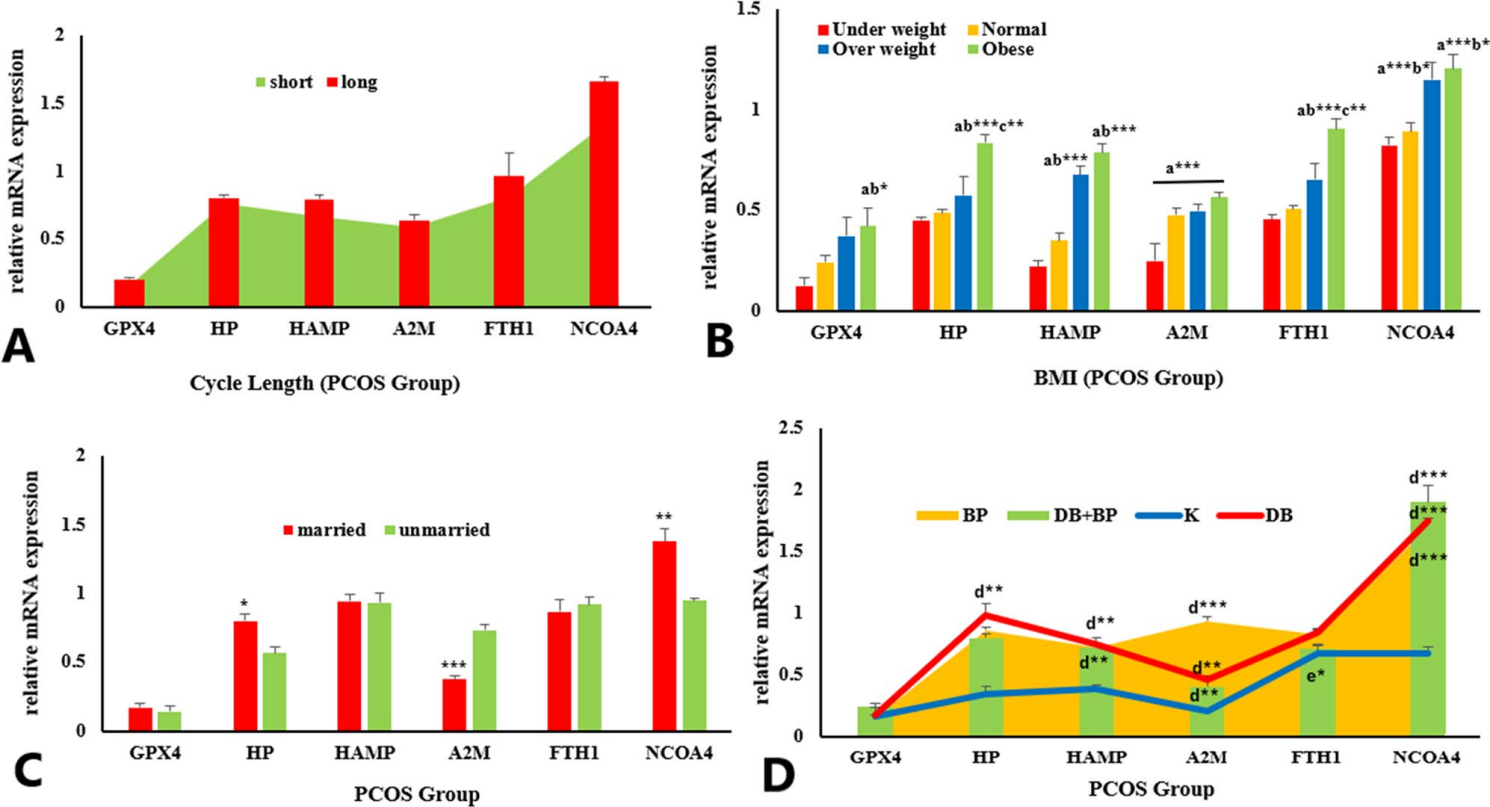
Relative expression of selected genes (GPX4, HP, HAMP, A2M, FTH1, NCOA4) in PCOS group showing comparison: between; short cycle length versus long cycle length (**A**), PCOS females with different BMI (**B**), married versus unmarried females (**C**), between PCOS females with other diseases (**D**). Data was expressed as Mean ± SEM. Data was compared using One-Way ANOVA followed by multiple comparison Tukey’s test. *P* < 0.05*, *P* < 0.01**, *P* < 0.001***. a = underweight versus other group, b = normal versus other group, c = overweight versus other group, d = kidney (K) versus other groups, e = diabetes (DB) versus other groups

Relative mRNA expression of GPX4, HP, HAMP, A2M, FTH1 and NCOA4 gene was downregulated in PCOS patients with short cycle length (< 28days) compared to females with long cycle length (> 35 days), although the difference was statistically non-significant (Fig. 3A).

### Body mass index (BMI)

Based on BMI, PCOS patients were sub-groups as underweight, normal, overweight and obese females and relative expression of selected genes was compared between these group. mRNA expression of GPX4 (*P* < 0.05), HAMP, FTH1 and HP (*P* < 0.001) gene were significantly higher (*P* < 0.05) in obese females compared to normal and underweight females. Even significant (*P* < 0.01) upregulation was observed in HP and FTH1 expression of obese group compared to overweight group. Relative expression of A2M gene was significantly (*P* < 0.05) increased in normal, overweight and obese group compared to underweight PCOS females. Similarly, NCOA4 expression was higher in overweight and obese female group compared to normal (*P* < 0.05) and underweight (*P* < 0.001) females (Fig. 3B).

### Marital status

Relative expression of selected genes was compared between married and unmarried PCOS females. mRNA expression of HP (*P* < 0.05) and NCOA4 (*P* < 0.01) was increased significantly while expression of A2M gene was decreased significantly (*P* < 0.001) in married women compared to unmarried women of PCOS group. Whereas, GPX4, FTH1 and HAMP displayed non-significant variation between married versus unmarried PCOS females (Fig. 3C).

### With other medical complications

Different medical complaints/diseases were observed in PCOS females. Based on commonly observed complications (kidney, diabetes and BP) PCOS females were further divided into sub-groups and mRNA expression of selected genes was compared between these groups. Relative expression of HP (*P* < 0.01) A2M (*P* < 0.01), NCOA4 (*P* < 0.001) and HAMP (*P* < 0.01, *P* < 0.05) gene was significantly downregulated in PCOS female with complaint of kidney disease compared to PCOS females with diabetes, BP and both with diabetes and BP. Similarly, expression of A2M and FTH1 gene was significantly upregulated in diabetic PCOS females compared to females with complaint of BP (*P* < 0.001) and both diabetes and BP (*P* < 0.05). other groups showed non-significant differences between one another (Fig. 3D).

### Biochemical assays

Biochemical parameters including serum peroxidase activity (POD), lipid peroxidation (TBARS), ferritin levels and total protein were estimated in control and PCOS patients.

### Peroxidase activity (POD)

**Fig. 4:**
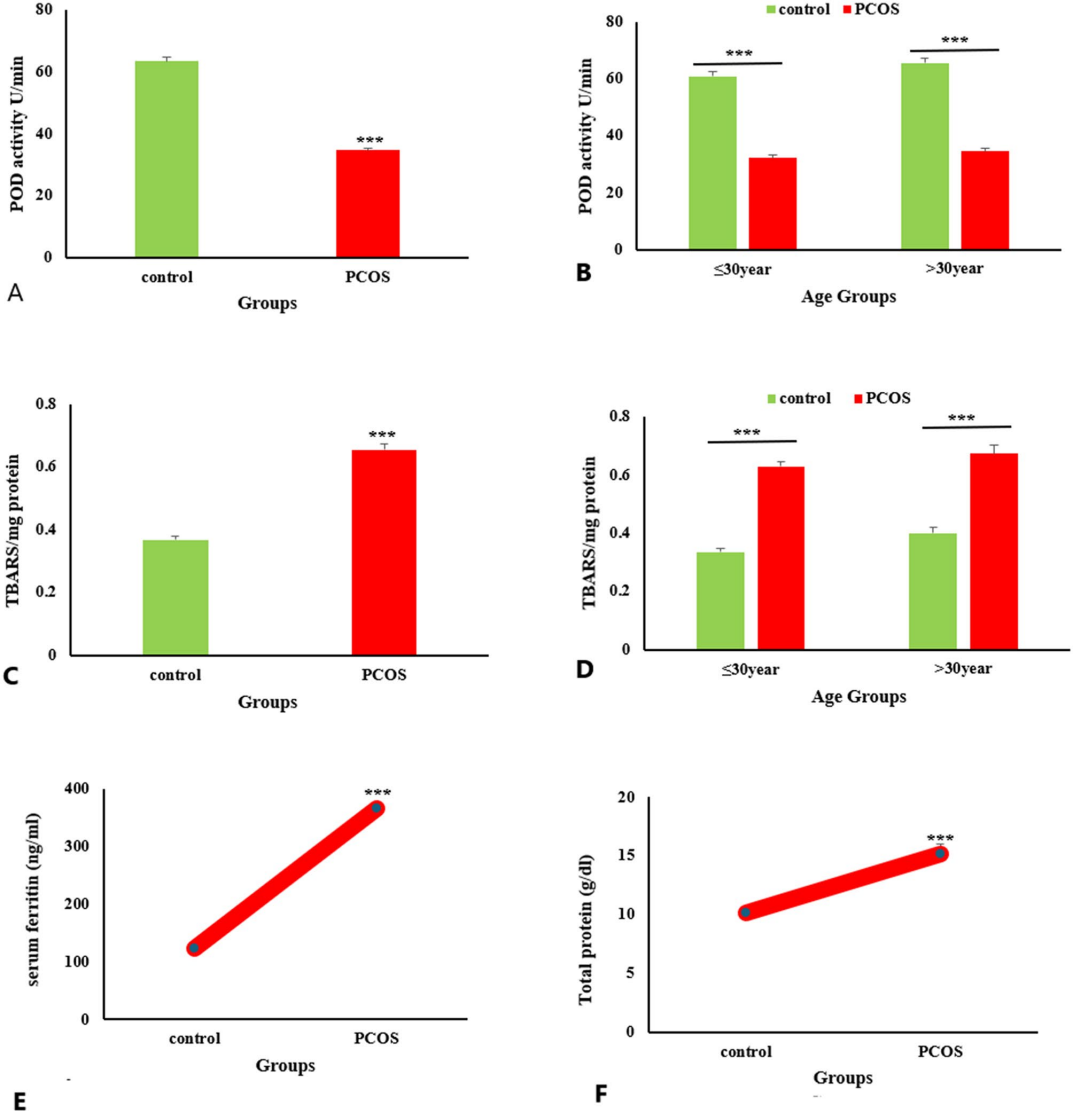
Biochemical assay of control and PCOS group showing comparison; POD activity between PCOS versus control (**A**), POD activity between PCOS versus control with different age group (**B**), lipid peroxidation (TBARS) between PCOS versus control (**C**), TBARS between PCOS versus control with different age group (**D**), serum ferritin between PCOS versus control (**E**), serum protein between PCOS versus control (**F**). Data was expressed as Mean ± SEM. Data was analyzed using student’s t-test. *P* < 0.001***

Serum POD activity was significantly reduced (*P* < 0.001) in PCOS females compared to healthy control subjects (Fig. 4A). POD activity in ≤ 30year age PCOS females was significantly lowered (*P* < 0.001) versus control subjects of similar age group. Likewise, POD activity was reduced (*P* < 0.001) in PCOS patients of > 30year age group versus control females with same age group (Fig. 4B).

### Lipid peroxidation (TBARS assay)

Elevated levels of lipid peroxidation (TBARS) was observed in PCOS patients compared to the healthy control females (Fig. 4C). TBARS was higher in ≤ 30year age of PCOS females versus control females of same age. Likewise, TBARS was increased in > 30years age of PCOS females compared to control females of similar age group (Fig. 4D).

### Serum ferritin and total protein

Levels of serum ferritin and total protein were significantly (*P* < 0.001) higher in PCOS patients compared to healthy control subjects (Fig. 4E and F).

### Correlation

**Fig. 5:**
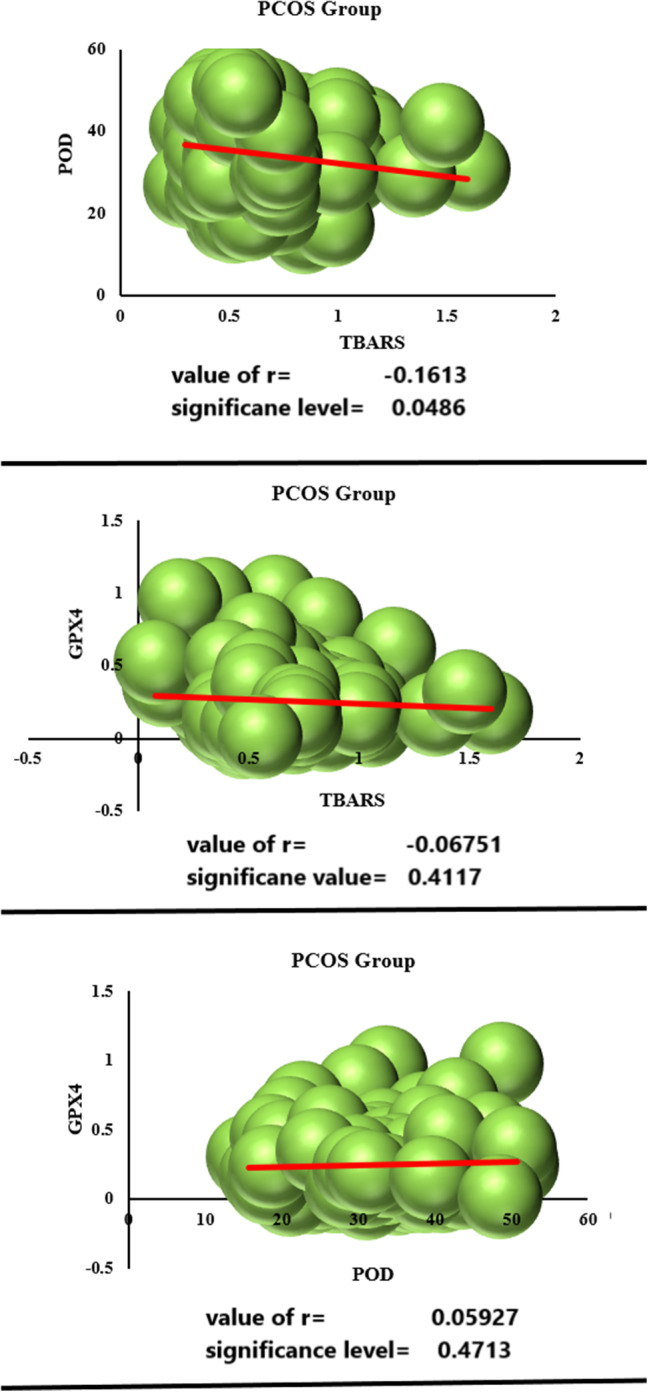
Pearson correlation coefficient was calculated in group of PCOS females showing; POD activity versus TBARS (**A**), mRNA expression of GPX4 versus TBARS (**B**), mRNA expression of GPX4 versus POD activity (**C**)

Pearson correlation coefficient was calculated between different parameters of PCOS group. Significant negative Pearson coefficient correlation was observed between POD activity versus TBARS (*P* < 0.05) activity in PCOS females (Fig. 5A). However, statistically non-significant negative correlation coefficient was found between GPX4 expression versus TBARS activity (Fig. 5B). Positive although non-significant correlation was observed between GPX4 expression versus POD activity in females of PCOS group (Fig. 5C).

## Discussion

The underlying mechanism of iron overload and its negative effect on female reproductive function is still ambiguous. Present study investigates the association of iron overload-induced ferroptosis with lipid peroxidation and genes relate to iron metabolism in females diagnosed with PCOS. Recognition of iron overload in PCOS patients is one of the facts about etiology of disease [16] as ferritin protein is a clear demonstrator of body iron status [11]. Altered patterns of various proteins related to iron metabolism were detected in PCOS patients advocating the manifestation of iron overload [26, 29]. Ferritin may act as a potential indicator for early screening of PCOS. Likewise, high levels of serum ferritin were observed in PCOS females which ultimately trigger ferroptosis and induce ferritinophagy.

Different genes related to iron metabolism were investigated to undrape this mechanism. Reduced HAMP gene expression was observed in PCOS females of current study was one possible reason to increase the serum ferritin levels as hepcidin is a strong regulator of iron homeostasis [16]. High iron content with decreased hepcidin level was reported in PCOS patients [30]. Hepcidin has strong efficacy to bind with ferroportin and α2-macroglobulin thus restricted the entry of iron into plasma [31, 32]. Expression of A2M gene was downregulated in PCOS patients of present study. Possibly HAMP and A2M gene acted synergistically to deregulate ferroportin hence elevate the accessibility of free iron. Moreover, haptoglobin protein binds to hemoglobin more effectively, hence decreased levels of plasma HP may depict the excessive availability of free hemoglobin and subsequently leading to iron overload [33]. In accordance, PCOS females of current research showed downregulation in mRNA expression of HP gene. Present research suggested that altered expressions of HAMP, HP and A2M genes might have contributed to induce iron overload in PCOS patients. To better understand the underlying mechanism association between ferroptosis and lipid peroxidation has been explored. Ferroptosis is an iron-dependent lipid peroxidation type of oxidative cell death, which may impair cell viability or may induce mitochondrial dysfunction. The whole process is facilitated by FTH1-NCOA4 complex, the cargo receptor of ferritin [34]. Expression of FTH1 was downregulated while NCOA4 was upregulated in PCOS females of present study. In ferritinophagy, NCOA4 binds with FTH1, and transport ferritin to lysosome for degradation hence facilitate the free iron release [35]. Although this FTH1-NCOA4 complex behave differently in different diseased conditions. Increased level of FTH1 enhances the degradation of ferritin hence promote ferroptosis [10, 36, 37]. Knockdown of NCOA4 reduces bioavailable free intracellular iron, restrict ferritinophagy, stop lipid peroxidation and limit ferroptosis [38]. In parallel the current study presented high levels of ferritin, low expression of FTH1, upregulation of NCOA4 along with elevated levels of lipid peroxidation and reduced POD activity in PCOS patients. High expression of NCOA4 and low of FTH1 was reported in human granulosa cells suggesting increased ferroptosis [39]. Subsequently high ferritin may increase the accumulation of lipid ROS resulted in membrane damage and ultimately to cell death [40]. In addition, expression of GPX4, the gatekeeper and inhibitor of ferroptosis [20], was significantly downregulated in PCOS patients of present study. Low expression of GPX4 would trigger the lipid ROS generation [41] and suppress the peroxidase activity. Therefore, the impact of iron overload not only affects the iron regulatory mechanism, but the redox status of the cell would also be sacrificed.

Different demographic parameters have been investigated to explore the possible association between age, cycle length and BMI with expression of selected genes in current research. Aging is one possible factor influencing gene expression, and it may be attributed to restricted physical activities which increase the susceptibility to disease risk [42]. Animal studies have suggested the association of aging with almost 75% of genes [43]. In present study differential expression of selected genes was observed in < 30year age versus < 30year age group of PCOS patients. Length of menstrual cycle in PCOS females has no significant association with expression of selected genes. Although changes have been observed in cellular configuration and expression of various genes. Possibly large-scale study or cohort study may look closer to find significant association between gene expression and menstrual cycle length, as the present study comprised total of 150 PCOS females. Obesity is another major risk factor for human health. Profound differences in expression of selected genes were observed in overweight and obese PCOS females compared to normal and underweight PCOS females. Inflammation and oxidative stress are commonly observed in obesity. Possibly both the factors induces DNA damage and might impair the repair machinery of DNA [44]. Finding of present study has postulated a strong association between selected genes and BMI of PCOS females and proposed obesity as one of the candidate which can risk the genomic integrity. Further studies will be required to detect DNA damage and to explore the repair mechanism in obese and overweight PCOS patients. This could be potentially useful in risk assessment between obesity and PCOS. Moreover, majority PCOS females in current study presented with complaint of diabetes, hypertension, or suffering from both in addition to PCOS. Women with PCOS are at risk of getting diabetes, obesity, and high BP [45–47], which coincides with the finding of current study. Present study provides evidence for kidney illness in PCOS females. Limited efforts have been made to find link; however, few studies suggested a risk of kidney diseases in the PCOS diagnosed patients [48, 49].

In conclusion present study suggested the involvement of iron overload in promotion of ferroptosis followed by restricted function of antioxidants and altered function of iron regulatory mechanism which greatly increase the lipid peroxidation and production of lipid ROS, resulted in oxidative stress ultimately trigger the iron dependent cell death known as ferroptosis.

### Study limitations

Although the present study opened a new avenue for diagnosis of PCOS based on iron related metabolism and ferroptosis, still some limitations have been observed. Possibly large sample size and cohort study will better explain the association between iron overload and PCOS progression. Comparison of PCOS females with other medical complication and PCOS females with no other medical complaints would describe the complete association of PCOS etiology with risk factors. But unfortunately, our study size is not enough to describe this parameter. In addition, evaluation of dietary iron intake, total iron levels and TIBC will give complete picture of current iron status of PCOS patients. Moreover, expression of inflammatory markers will better display the underlying mechanism of PCOS etiology.

## Data Availability

No datasets were generated or analysed during the current study.
